# Evaluation of the *SD BIOLINE HIV/syphilis* Duo assay at a rural health center in Southwestern Uganda

**DOI:** 10.1186/1756-0500-7-746

**Published:** 2014-10-22

**Authors:** Daniel Omoding, Victoria Katawera, Mark Siedner, Yap Boum

**Affiliations:** Epicentre Mbarara Research Centre, Mbarara, Uganda; Mbarara University of Science and Technology, Mbarara, Uganda; Massachusetts General Hospital and Harvard Medical School, Boston, USA

**Keywords:** HIV, Syphilis, Duo kit

## Abstract

**Background:**

Point-of-care tests have the capacity to improve healthcare delivery by reducing costs and delay associated with care. A novel point-of-care immunochromatographic test for dual diagnosis of both HIV and syphilis by detecting IgG, IgM and IgA antibodies to HIV, and specific and recombinant *Treponema pallidum* antigens has recently been developed, but has not been evaluated in rural field settings. We evaluated the performance of the SD Bioline Syphilis/HIV Duo (Duo) assay at a healthcare center in rural Uganda.

**Methods:**

A convenience sample of pregnant women attending Kinoni Health Centre IV from March to May, 2013 was enrolled. Venous blood was collected and centrifuged for plasma isolation. Samples were tested with the Duo assay and compared with the Treponema pallidum hemaglutination assay and paired HIV rapid antibody tests as the reference standards. The ease of use and time required for the Duo assay were also assessed by laboratory technicians.

**Results:**

Two hundred twenty women were enrolled with a mean age of 25.00 years (SD 5.41). The sensitivity and specificity of the Duo assay were 100% (95% CI 79.0 – 100%) and 100% (95% CI 97.6 – 100.0) respectively, for syphilis, and, 100% (75.9 – 100%) and 99.5% (96.8 – 99.9%) respectively, for HIV. The duo kit was found to be faster and easier to use than the current HIV and syphilis testing techniques.

**Conclusion:**

The sensitivity and specificity of the SD Bioline HIV/Syphilis Duo test were excellent in a field setting in Uganda. The Duo assay should be further evaluated in alternate populations and with point-of-care specimens (e.g. whole blood from finger stick specimens), but shows promise as a tool for improved HIV and syphilis surveillance, diagnosis, and treatment in field settings.

## Background

Syphilis is a curable infectious disease, estimated to infect 12 million people annually, [1], including an estimated 1.4 million pregnant women, [2]. The prevalence is highest in low income settings, where syphilis prevalence among pregnant women ranges as high as 17% [1]. Untreated maternal syphilis during pregnancy is a particularly critical public health problem because the infection can cause adverse birth outcomes in 60-80% of children born to infected women, including stillbirth, premature birth, neonatal death, low birth weight, congenital syphilis and disability [1]. Early screening for and treatment of syphilis during pregnancy can effectively prevent adverse birth outcomes [3]. However, fewer than half of pregnant women receive ante-natal syphilis testing in many low resource settings, [4], likely due in part to cost and infrastructure required of classic syphilis assays [5].

Similarly, while an estimated 1.5 million pregnant women giving birth each year are HIV infected, only approximately 35% are tested for HIV [6]. Early accurate diagnosis to aid proper treatment and management is a cornerstone of prenatal care and central to effective prevention of mother-to-child transmission (MTCT) of HIV [6]. In addition, syphilis infection during pregnancy greatly increases the risk of MTCT of HIV [7].

The World Health Organization (WHO) has recommended the dual elimination of MTCT HIV and syphilis, with new strategies and integrated monitoring and evaluation activities [8]. Screening for HIV and syphilis are recommended as part of a comprehensive dual elimination strategy [9]. Moreover, integrating rapid syphilis screening and HIV testing for pregnant women has been found to be feasible, cost-effective, and effective in preventing MTCT of syphilis and HIV [10]. However, while many countries have adopted policies recommending integration, comprehensive implementation has not been achieved [10].

Conventionally, conducting concurrent HIV and syphilis tests require two separate diagnostic modalities: a point-of-care HIV serology assay and a laboratory-based non-treponemal assay or, where available, a second point-of-care syphilis assay. Recently, the SD Bioline Syphilis/HIV Duo Assay (Duo), a rapid, lateral flow assay has been developed to concurrently test for both syphilis and HIV. The assay is designed to detect IgG, IgM and IgA antibodies of both syphilis (treponemal serology) and HIV. The test costs $2US dollars per kit, can be performed by non-laboratory personnel, and takes approximately 20 minutes to complete. Incorporation of both tests in a single kit has potential advantages, including decreased needle use and blood volume, eliminating the need for laboratory personnel or infrastructure, decreased time to results, and lowering diagnostic testing costs. However, the dual assay has not been tested in rural field settings.

We evaluated the performance of the Duo assay at a pre-natal clinic in rural Uganda. We used the *Treponema pallidum* haemaglutination assay (TPHA) and the Uganda HIV screening algorithm [11] as reference standards. The goal of this study was to assess the diagnostic accuracy of this test using venous samples prior to evaluation of its feasibility and accuracy as a point-of-care tool.

## Methods

We conducted a cross-sectional study in Kinoni Health Centre IV, Mbarara, Uganda, from March to May, 2013. Venous blood samples were collected into EDTA tubes from a convenience sample of pregnant women attending clinic for routine prenatal clinical care.

The study was approved by the faculty research ethics committee (FREC) Mbarara University of Science and Technology and the study participants provided written informed consent.

All procedures were completed by laboratory technicians at the Kinoni Health Center. Whole blood samples were centrifuged at 1300rcf for 5 minutes and plasma was separated. We followed the manufacturer’s instructions to test plasma samples with the Duo assay (Standard Diagnostics, Inc. Yongin, Gyeonggi, South Korea). Briefly, 20 μl of plasma and three drops of buffer were added to the sample area. Results were read by two independent laboratory technicians after 20 minutes.

For our reference standards we used the Treponemal pallidum hemagluttination assay (TPHA) for syphilis and the series method of the national HIV testing algorithm [12], for HIV. In brief we tested all samples serially with 1) the Determine HIV-1/2/O assay (Abbott Laboratories, Abbott Park, IL), 2) the HIV 1/2 Stat-Pak Ultra Fast (Chembio Diagnostic Systems, Medford, NY) and 3) the Uni-Gold Recombinant HIV-1/2 (Trinity Biotech, Bray, Ireland) assay. A negative first assay was considered negative. A positive first assay was followed by a second assay for confirmation. A third test was performed for discordant results between the first two tests.

We calculated the sensitivity and specificity, and corresponding 95% confidence intervals (95% CI) of both components of the Duo assay using Stata Version 12.0. We also calculated the frequency of indeterminate results. We also assessed the ease of use and time required for the Duo assay.

## Results

Two hundred twenty women were enrolled with a mean age of 25.00 (SD 5.41). Of the 220 study participants’ samples tested, antibodies against *T. pallidum*, HIV, and, both were detected in 19 (8.6%) (Table 1), 16 (7.3%) (Table 2) and 3 (1.4%), respectively. The sensitivity and specificity of the Duo kit (Table 3) was 100% (95% CI 79.0 – 100%) and 100% (95% CI 97.6 – 100%) for syphilis, and, 100% (95% CI 75.9 – 100) and 99.5% (95% CI 96.8 – 99.9%) for HIV. The Duo kit took approximately 25 minutes to perform in the laboratory. The test results were read by two different laboratory technicians and they both found it easy to use by way of assessing amount of sample manipulation involved. There was 100% inter-reader agreement for both the HIV and syphilis results.

**Table 1:** **Positive and negative tests of** ***SD BIOLINE HIV*** **/** ***syphilis*** **Duo kit for syphilis**

		TPHA test		
		Positive	Negative	
**HIV-syphilis duo results**	Positive (%)	19	0	19 (8.6)
	Negative (%)	0	201	201 (91.4)
	**Total (%)**	19	201	**220 (100)**

**Table 2:** **Positive and negative tests of** ***SD BIOLINE HIV*** **/** ***syphilis*** **Duo kit for HIV**

		HIV algorithm		
		Positive	Negative	
**HIV-syphilis duo results**	Positive (%)	16	1	17 (7.7)
	Negative (%)	0	203	203 (92.3)
	**Total (%)**	16	204	**220 (100)**

**Table 3:** **Summary of the validity and reliability of** ***SD BIOLINE HIV*** **/** ***syphilis*** **Duo kit**

	Sensitivity (95% CI)	Specificity (95% CI)	PPV (95% CI)	NPV (95% CI)
Syphilis (%)	100.0 (79.1 - 100.0)	100.0 (97.7 - 100.0)	100.0 (79.1 - 100.0)	100.0 (97.7 - 100.0)
HIV* (%)	100.0 (75.9 - 100.0)	99.5 (96.9 - 99.9)	94.1 (69.2 - 99.7)	100.0 (97.7 - 100.0)

TPHA – Treponema pallidum hemagglutination assay; HIV algorithm - Determine, Statpak and UniGold tests run serially; Duo kit – *SD BIOLINE HIV/syphilis* duo kit.

*HIV-syphilis results were obtained from TPHA and HIV algorithm results.

## Discussion

The sensitivity (100%) and specificity (100%) for syphilis, and sensitivity (100%) and specificity (99.5%) for HIV, of the Duo HIV and syphilis lateral flow assay were excellent when tested with a population of women attending routine maternal care in southwestern Uganda. These data provide proof of principle for feasibility and accuracy of this assay in similar settings. If our findings are corroborated in larger studies and with point-of-care specimens, they could support broad use of the assay to facilitate diagnosis of both HIV and syphilis in resource-constrained settings with minimal human resource or laboratory capacity.

Prior studies have shown similar results. A large (n = 2336), multinational study demonstrated a sensitivity of 99.91% and 99.67% and specificity of 99.67% and 99.72% for HIV and syphilis, respectively [13]. A notable difference between that study and ours was that the assay was performed in centralized laboratories. Two other recent reports from California, USA and England using archived specimens noted sensitivities and specificities above 99.5% for both assays [14, 15]. Our study contributes a validation of the assay at a peripheral health center in a resource-limited setting and adds to the growing evidence in support of the high accuracy of the assay across various settings.

Although we performed our tests in a laboratory setting, the assay was found to be quicker (approximately 25 minutes), easier to use, and cheaper (approximately $2USD versus $2USD for TPHA and $3-10USD for the HIV algorithm) than the current techniques for HIV and syphilis testing/screening. Moreover, running the tests separately adds a requirement for additional time, costs, and human resources. Lastly, we found no indeterminate results and inter-reader agreement was found to be 100%. Further evaluation of these results with whole blood specimens will be important to confirm the feasibility and ease of use of the assay.

The HIV prevalence reported in our study is comparable to that reported by the Uganda AIDS Indicator Survey (8.3%, [16]). In contrast, we estimated a considerably lower prevalence of syphilis than other national estimates [17]. This difference might have been due to local differences in syphilis rates, or recent improvement in syphilis testing and treatment programs in Uganda [10]. In spite of the similar or lower prevalence obtained in this study, the HIV, syphilis and HIV-syphilis co-infection rates are still alarming and signal an urgent need to improve efforts to prevent, detect and treat syphilis and HIV in similar populations.

The most important limitation of this study was the use of venous blood which was centrifuged and tested in a laboratory as opposed to point-of care whole blood specimens obtained from a figure prick. We did so to determine the best-case diagnostic accuracy of the assay prior to a more extensive point-of-care evaluation of test feasibility, ease of use, and accuracy. Even without point-of-care evaluation, our results do offer promising data on the use of the Duo kit in centers that do have laboratory capacity, based on its ease of use, time to results, and accuracy. Our study was also performed in a single center and on a specific patient population. Larger, multicenter studies will help broaden the generalizability of our results.

## Conclusion

The continuing worldwide syphilis and HIV epidemics and their resultant impact on both maternal and child health warrants increased attention to prevention and delivery of high quality syphilis and HIV care. Rapid, low-cost assays that can be incorporated into public health programs in resource-constrained settings are an important step in this process. We found that the Duo kit had excellent performance with venous specimens from women accessing prenatal care in rural Uganda. The test should be further evaluated with point-of-care whole blood specimens obtained from a finger prick and in broader populations, but shows promise as an additional tool for improved HIV and syphilis surveillance, diagnosis, and treatment in similar settings.
